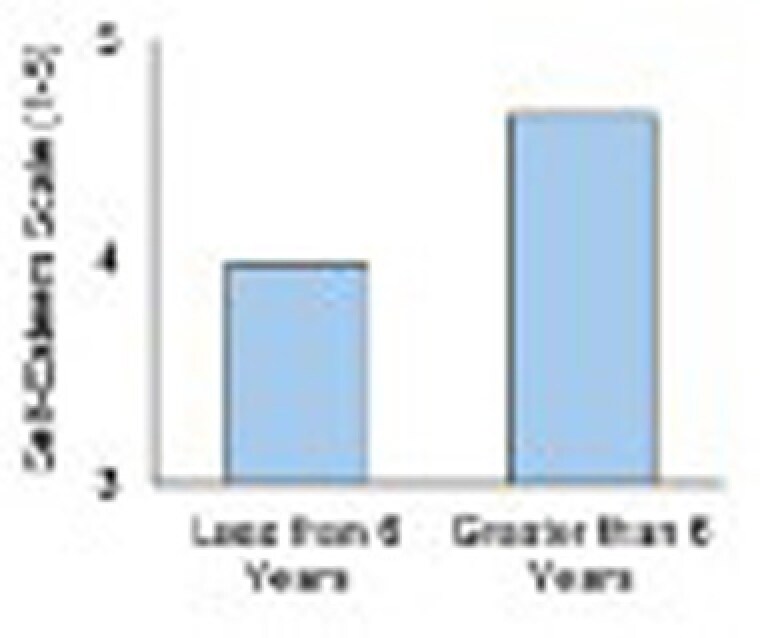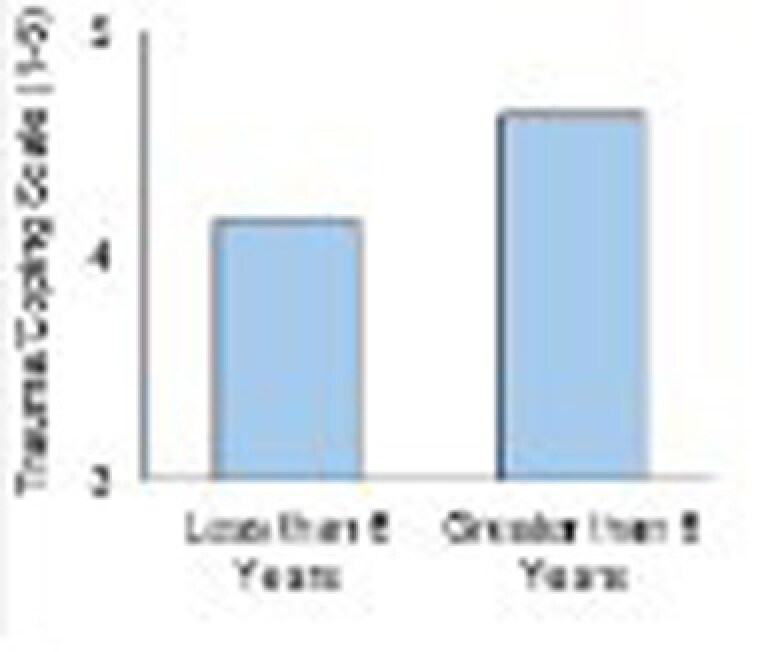# 630 Efficacy of Child and Teen Support Groups After Traumatic Burn Injury

**DOI:** 10.1093/jbcr/iraf019.259

**Published:** 2025-04-01

**Authors:** Tanya Sorkin, Linda Garcia, Shu-Sha Guan, Yekaterina Vaganyan, Michelle Escamilla Valladares

**Affiliations:** Children’s Burn Foundation; Children’s Burn Foundation; California State University, Northridge; California State University, Northridge; Children’s Burn Foundation

## Abstract

**Introduction:**

Upon hospital discharge from traumatic burn injuries, children, teens and caregivers require continuous psychological interventions for full recovery. Participation in burn camps and school reentry contribute greatly to peer support and integration back into community.

Ongoing support groups are offered for child burn survivors (ages 6-12), teens (ages 13-18), siblings and parents. This unique model allows for peer support, developing healthy coping strategies, sharing burn trauma related experiences, changing developmental milestones, and integration into community.

The goal of this study was to assess effectiveness of support groups in promoting well-being for pediatric burn survivors and their families.

**Methods:**

Parents were surveyed for this prospective study whose children were treated for burn injuries at one of five local hospitals.

Respondents (22) out of 62 families participated with no drop-out. Respondents answered 49 questions (scaled 1-5) and 10 open ended questions developed by support group facilitators about their children’s health, quality of life, self-esteem, trauma, coping, communication, social support, relationships, behaviors, and support group effectiveness. Study utilized existing measures: NIH PROMIS: Parent Proxy – Global Health, Meaning and Purpose, Family Relationships; Modified scales Rosenberg Self-Esteem 1965; Strengths and Difficulties Questionnaire – Impact Supplement Goodman, 2001; Husky et al. 2020.

**Results:**

All participants were Latina/o/x, average age was M = 42.64 (SD = 9.76), and child burn survivor age M = 14.20 (SD = 5.03). Average annual family income = $69,181. Most parents are married and work full-time.

Majority of families participated in both the school-age and teen groups. Average years of participation was M = 6.32 years (SD = 3.86 years). Study found those who participated for 6 years or more reported significantly higher child burn survivor self-esteem (t(17) = 2.51, p =.022) and trauma coping (t(17) = 2.48, p =.024). Higher self-esteem and trauma coping may have important implications for global health, quality of life, communication, and social support. Participants who felt more supported in group looked forward to attending. Children whose behavior improved were more accepting of their injuries.

**Conclusions:**

Consistent and long-term (six years or more) participation in support groups provides emotional healing, post traumatic growth and improves outcomes and quality of life for child and teen burn survivors.

**Applicability of Research to Practice:**

Burn survivors face challenges beyond physical recovery. Long term interventions are necessary to thrive into adulthood. Children are especially at risk due to early trauma, physical changes and social pressures. Continuous participation in support groups provides psychological healing that is part of the full recovery process.

**Funding for the Study:**

Foundation Funding